# Hypertension and risk of cardiovascular disease. A major problem

**DOI:** 10.1186/1824-7288-41-S2-A48

**Published:** 2015-09-30

**Authors:** Silvio Maringhini

**Affiliations:** 1 U.O.C. Nefrologia Pediatrica, Ospedale “G. Di Cristina”, A.R.N.A.S. Civico, 90100 Palermo, Italy

## 

Cardiovascular diseases (CVD), namely coronary artery disease and cerebrovascular accidents, are the main cause of mortality and morbidity worldwide [1]. Risk factors for CVD in adults are: hypertension, diabetes, hyperlipidemia, hyperuricemia, microalbuminuria, overweight and obesity; over-eating, physical inactivity, tobacco smoking, exaggerated alcohol consumption, represent risk habits [2]. The WHO has promoted a program of prevention of CVD in adults [3]. It has been proved that risk factors for CVD are already present in childhood [4]; genetic and environment factors play a role and early intervention may produce a benefit [5]. Hypertension is present in a consistent percentage of adults and is related to high mortality rate. Criteria used for diagnosis of hypertension in children are more restrictive but it has been proved that adults with hypertension have high values of blood pressure in childhood [6]. Blood pressure measurement in children should been done according to standard procedures; ambulatory blood pressure (ABP) may be a better predictor of adverse cardiovascular events than office BP (OBP) [7]. Vascular biomarkers, which are parameters of subclinical cardiovascular disease, could increase the estimation of the individual cardiovascular risk and can be measured by non-invasive techniques [8]. Familial hypertension, low birth weight, high BMI high sodium consumption, reduced physical activity in childhood are risk factors for hypertension in adulthood. Renal insufficiency is often associated with hypertension and it is well known that uremia represents an increased risk for CVD, but congenital renal disease may produce hypertension and CVD before glomerular filtration rate is reduced [9]. Pediatricians have a crucial role in preventing hypertension and CVD [10].
